# Supplementary material for: Long-term root electrotropism reveals habituation and hysteresis
Source: Plant Physiol. 2023 Dec 29;194(4):2697–708. doi: 10.1093/plphys/kiad686 (PMC10980514; doi:10.1093/plphys/kiad686)
Supplement: kiad686_Supplementary_Data [file kiad686_supplementary_data.pdf]

Long-term root electrotropism reveals habituation and hysteresis

Maddalena Salvalaio[1] and Giovanni Sena[1*]

[1] Department of Life Sciences, Imperial College London, London, SW7 2AZ, UK
[*] Corresponding author: g.sena@imperial.ac.uk

Supplemental Information

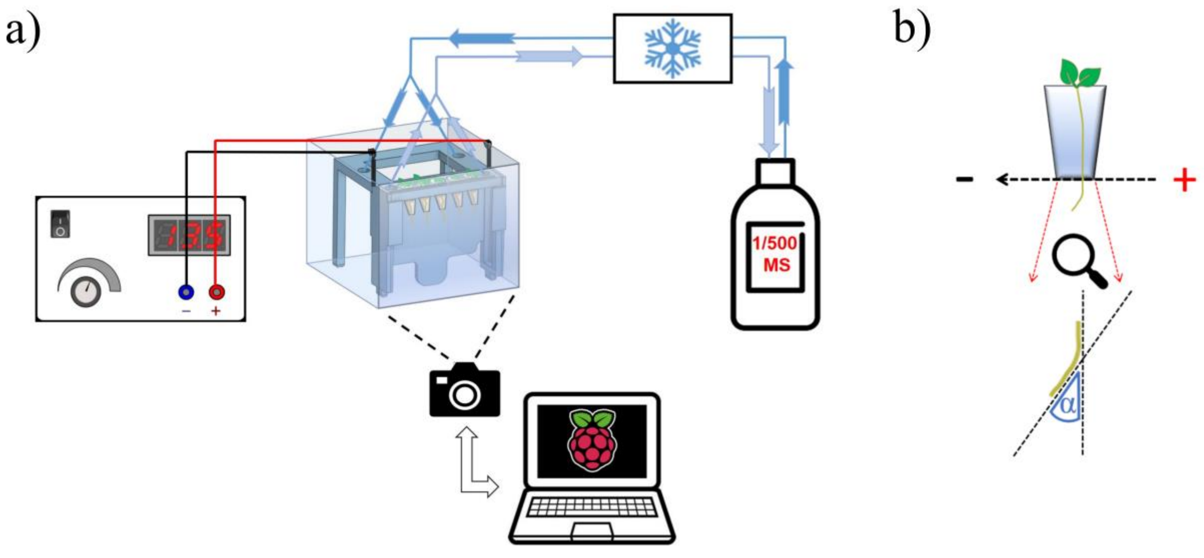

Supplemental Figure 1. Root electrotropism assay. **a**, Schematic of the V-box connected to the power supply and the medium circulation system; **b**, measured angle between the root tip and the gravity vector.

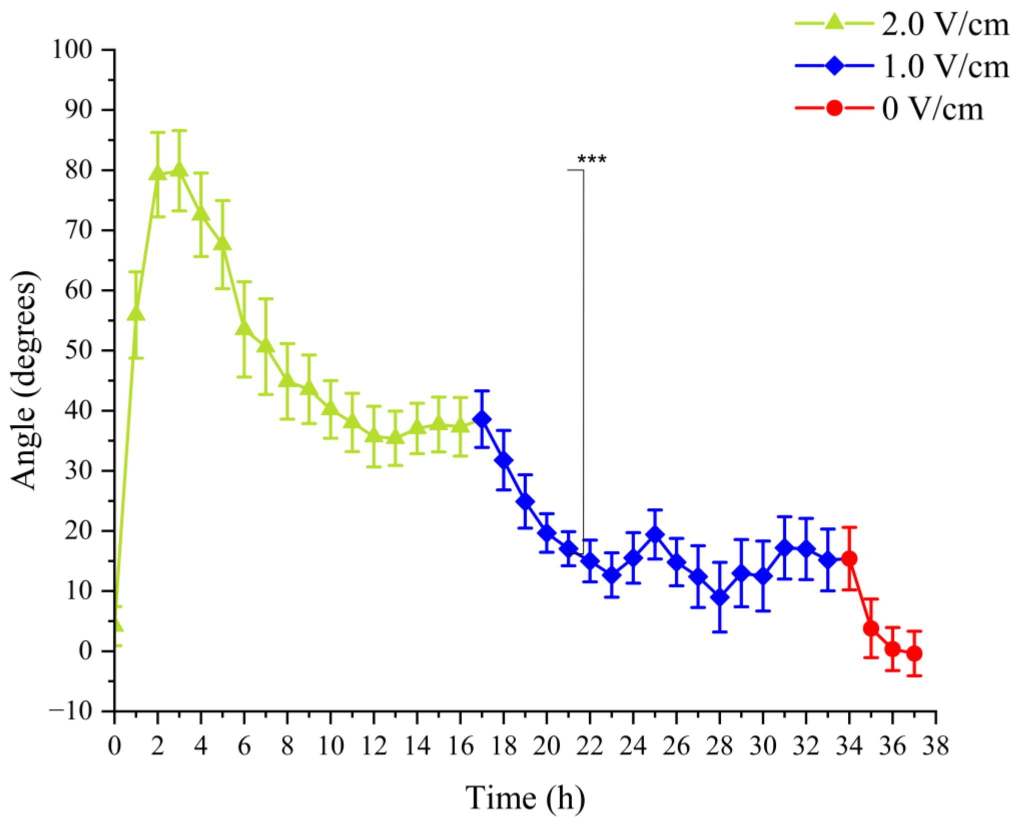

Supplemental Figure 2. Decrease of E-field intensity. Average WT root tip orientations relative to the gravity vector versus time when a change in EF intensity 2.0 V/cm → 1.0 V/cm → 0 V/cm (N=10, R=2) is applied; N=18, R=4. N, sample size; R, number of replicates. Error bars, s.e.m. *** $p < 0.001$ (Student's *t* test).
